# *Ex vivo* multiscale quantitation of skin biomechanics in wild-type and genetically-modified mice using multiphoton microscopy

**DOI:** 10.1038/srep17635

**Published:** 2015-12-03

**Authors:** Stéphane Bancelin, Barbara Lynch, Christelle Bonod-Bidaud, Guillaume Ducourthial, Sotiris Psilodimitrakopoulos, Petr Dokládal, Jean-Marc Allain, Marie-Claire Schanne-Klein, Florence Ruggiero

**Affiliations:** 1 Laboratory for Optics and Biosciences, Ecole Polytechnique, CNRS, INSERM U1182, 91128 Palaiseau Cedex, FRANCE; 2 Solids Mechanics Laboratory Ecole Polytechnique, CNRS, Mines ParisTech, 91128 Palaiseau Cedex, FRANCE; 3 Institut de Génomique Fonctionnelle de Lyon, ENS-Lyon, CNRS UMR 5242, Université Lyon 1, 46 Allée d’Italie, 69364 Lyon, cedex 07 France; 4 Centre for Mathematical Morphology, MINES ParisTech, PSL Research University, 35 rue St Honoré, 77300 Fontainebleau, France

## Abstract

Soft connective tissues such as skin, tendon or cornea are made of about 90% of extracellular matrix proteins, fibrillar collagens being the major components. Decreased or aberrant collagen synthesis generally results in defective tissue mechanical properties as the classic form of Elhers-Danlos syndrome (cEDS). This connective tissue disorder is caused by mutations in collagen V genes and is mainly characterized by skin hyperextensibility. To investigate the relationship between the microstructure of normal and diseased skins and their macroscopic mechanical properties, we imaged and quantified the microstructure of dermis of *ex vivo* murine skin biopsies during uniaxial mechanical assay using multiphoton microscopy. We used two genetically-modified mouse lines for collagen V: a mouse model for cEDS harboring a *Col5a2* deletion (a.k.a. *pN* allele) and the transgenic *K14-COL5A1* mice which overexpress the human *COL5A1* gene in skin. We showed that in normal skin, the collagen fibers continuously align with stretch, generating the observed increase in mechanical stress. Moreover, dermis from both transgenic lines exhibited altered collagen reorganization upon traction, which could be linked to microstructural modifications. These findings show that our multiscale approach provides new crucial information on the biomechanics of dermis that can be extended to all collagen-rich soft tissues.

Connective tissues such as skin, tendon, cornea and bones are made of more than 90% (dry weight) of extracellular matrix (ECM) proteins, collagens being by far the predominant component^1^. These structural proteins form a superfamily of proteins that show a remarkable diversity in molecular and supramolecular organization and their hierarchical organization in ECM play a unique role in maintaining the structural integrity and biological function of most connective tissues. Fibrillar collagens form a subset of the collagen superfamily that is characterized by a long uninterrupted triple-helical domain and their capability to assemble into fibers. The fiber diameter and organization depend on tissues and provide mechanical support to residing cells. Defective or reduced amounts of fibrillar collagen molecules typically disrupt tissue mechanical properties and/or tissue cohesion by altering cell interactions with the surrounding ECM. Such changes in tissue biomechanics have a dramatic impact in development, wound repair and ageing (for review see^2^). As typical example, mutations in collagen V gene are responsible for the classic form of the Elhers-Danlos Syndrome (cEDS) which is a connective tissue heritable disorder mainly characterized by defective mechanics of skin and joints^3^.

The physiopathogenesis of cEDS is not completely understood. Skin is a complex multilayered tissue consisting of the superficial epidermis, the dermis, a collagen**-rich heterogeneous compartment that supports the skin and the deepest hypodermis made of adipose tissue. Collagen V is a minor component of skin that plays a crucial role in the control of collagen I fibrillogenesis. Collagen V exists in several molecular forms. In dermis, the predominant subtype is the heterotrimer [α_1_(V)]_2_α_2_(V) while the [α_1_(V)]_3_ homotrimer represents a minor molecular species^2^. Genetically-modified mice for collagen V have been generated and constitute good models for cEDS; the *Col5a2*^*pN/*+^ mice with an in-frame exon 6 deletion in *Col5a2*^4^,^5^ and the *Col5a1* knock-out mice^6^. Both showed abnormally stretchy skin. Analysis of the phenotype revealed that the collagen V heterotrimer controls the nucleation and the diameter of heterotypic fibers in skin and that a certain threshold of collagen V should be maintained for normal collagen fibrillogenesis^4^,^5^,^6^. In contrast, homotrimer was shown to form thin filamentous structures localized at the surface of collagen I fibrils that contribute to the stabilization of the epidermal-dermal interface. These studies underscored the regulatory role of collagen V in the matrix organization/architecture of dermis but its contribution to skin mechanics remains poorly defined.

Macroscopic mechanical response of normal skin shows an initial non-linear part, the so-called heel region, followed by a linear part and a saturation, initiating the rupture of the tissue^7^,^8^,^9^. Similar mechanical responses have been reported for most soft collagen-rich tissues, such as tendon^10^,^11^,^12^, dura-mater^13^, cornea^14^,^15^, aorta^16^,^17^. The link between microstructure evolution and mechanical response has been mostly investigated in the highly organized tendon, with various methods: polarized-light microscopy^18^, OCT^19^, multiphoton microscopy^12^,^20^, confocal microscopy^11^,^21^ and X-ray diffraction^22^,^23^. All these studies concluded that the heel region is associated to the uncrimping of the collagen fibers, and therefore to their alignment in the traction direction. In the linear part, the response of a tendon is a combination of the stretching of the fibers and of a sliding of the fibers with respect to each other^22^,^23^. Like for tendon, the mechanical response of skin has been interpreted as an alignment of the fibers in the heel region, followed by the stretching of the fibers in the linear part^24^,^25^. This has led to the so-called microstructural models^26^,^27^,^28^,^29^. However, only few papers have investigated experimentally this interpretation at all stretch levels in skin^24^,^25^. For further insight into the microscopic processes of complex disordered tissues such as skin, a continuous monitoring of skin microstructure during mechanical assays is required.

To address this issue, we have recently combined multiphoton microscopy (MPM) with traction assays^12^,^20^. MPM has emerged as a powerful technique to investigate the three-dimensional (3D) architecture of collagen-rich tissues such as skin^30^,^31^,^32^. Due to intrinsic optical sectioning, it is robust upon light scattering and offers deep penetration within tissues. MPM modes of contrasts include Two-Photon Excited Fluorescence (2PEF) and Second Harmonic Generation (SHG). SHG appears at exactly half the excitation wavelength and is specific for dense non-centrosymmetric media, such as fibrillar collagen^33^,^34^,^35^,^36^. SHG microscopy represents an effective structural probe of the micrometer-scale collagen organization in unstained tissues^20^,^37^,^38^,^39^,^40^. We have demonstrated the combination of this technique with mechanical assays in tendon, as an uniaxial tissue model^12^,^20^. Extension of this approach to more complex tissues has been reported on aorta^41^,^42^, fetal membrane^43^,^44^, bone^45^, nerve^46^,^47^ and heart valves^48^, illustrating the reorganization of the collagen network with the load. Finally, application to skin has been reported very recently and showed that the fibers align in the direction of traction in the heel region^49^. However, this study was limited to small stretching values and the skin behavior in the linear part has not been investigated.

In this study, we developed a multi-scale system to investigate the relationship between the microstructure of the dermis and its macroscopic mechanical properties at all stretch levels. Mechanical measurements were performed while imaging optical sections of *ex vivo* mouse skin using multiphoton microscopy and specific image processing based on mathematical morphology algorithms was implemented to analyze tissue microstructure and correlate it with stress/stretch relationship. We took advantage of well-characterized mouse strains in which expression of collagen V has been modified, the transgenic *K14-COL5A1*^50^ and the knock-in *Col5a2*^*pN/*+^ 4,5 mice, to unravel the role of collagen V in skin mechanics.

## Results

### Design of *ex vivo* murine skin samples

We first designed reproducible protocols to obtain *ex vivo* skin with well-preserved structure and suitable features for direct optical measurements. One month-old wild-type (WT), transgenic *K14-COL5A1* or heterozygous *Col5a2*^*pN/*+^ littermates with the same genetic background (129sv) were used (see Supplementary Tables S1–S6). Wild-type specimens (WT) were obtained from 129sv mice, by crossing transgenic mice *K14-COL5A1 (K14-COL5A1*^*WT*^) or *Col5a2*^*pN/*+^ heterozygous mice (*Col5a2*^+*/*+^). *Col5a2*^*pN/pN*^ mice died perinatally and, at this stage, the total surface of the skin of the back was too small and fragile to be used for this study. Back skin was dissected immediately after sacrifice of the mice and, for optimal optical measurements, depilated epidermis was removed as described^50^. The dermis is not homogeneous in structure and organization. It is sub-divided into two histologically distinct layers (Supplementary Fig. S1) whose mechanical properties are expected to be different. The thin superficial papillary dermis comprises loosely arranged thin collagen fibers and the deeper reticular dermis is composed of dense bundles of thick collagen fibers. The treatment efficiently removed epidermis and hair without altering the underlying dermis structure as judged with histological Herovici’s staining (Supplementary Fig. S1). Based on this observation and on literature considerations, stating that the epidermis contribution is negligible^51^,^52^,^53^, we considered that this protocol did not modify substantially the mechanical properties of the skin. These skin samples were stored in culture medium at 6° and used within five days for mechanical experiments (see Methods). We did not observe any significant difference between skin samples tested at days 3, 4 and 5 after sacrifice.

### Multiscale mechanical measurements on *ex vivo* murine skin

**Figure 1:**
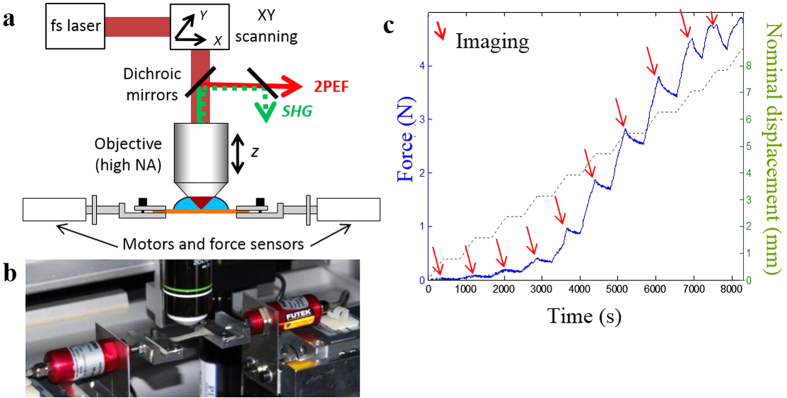
Experimental setup and protocol. (**a**) Scheme of the combined multiphoton microscope and traction device. 2PEF and SHG are detected in the backward direction; (**b**) View of the skin sample maintained under the microscope objective by two jaws fixed to two motors and two force sensors; (**c**) Experimental timetable showing stepwise increasing stretching of the skin sample (green dotted line) with continuous force measurement (blue straight line) and time-lapse imaging of immobile sample after each displacement step (red arrows).

We next implemented mechanical assays coupled with multiphoton microscopy to characterize the variation of collagen fibers organization in the *ex vivo* de-epidermilized skins under stretching. Skin samples were cut into a dog-bone shape to minimize side effects and attached to the traction device, which was inserted in place of the microscope stage in the multiphoton microscope (Fig. 1a,b). Skins were always oriented the same way, with traction along the head-tail axis and the papillary dermis facing the objective lens. Immersion gel was used to ensure optical contact with the objective lens and prevent skin dehydration during experiments. The samples were then increasingly stretched from their reference configurations at constant strain rate by steps of 0.05 strain, until they broke, usually at a stretch ratio around 1.5. Multiphoton imaging was performed at each step, while the motors were stopped to avoid skin movements during imaging. The same region was imaged at each strain step thanks to symmetrical traction on both sides of the sample and possible slight adjustment of the traction device position under the microscope. A typical loading path is displayed in Fig. 1c, along with force measurements that showed a continuous increase when the displacement was increased, interrupted by relaxations while the displacement was kept constant for imaging.

These experiments were carried out on skin samples from 1 month old WT (n = 25), *K14-COL5A1* (n = 9) and *Col5a2*^*pN/*+^ (n = 18) mice (see Supplementary Tables S1–S6). Similar mechanical assays were also performed without multiphoton imaging in a set of other mice skin samples to improve the statistics of mechanical data (WT (n = 7) and *K14-COL5A1* (n = 7) mice) (see Supplementary Tables S5–S6). No significant difference was observed on the mechanical properties (tangent modulus, length of the heel region, ultimate tensile stress and failure stretch ratio) between experiment under multiphoton microscope (with relaxation during imaging) and outside the microscope (without relaxation), meaning that the pauses during SHG imaging did not strongly affect the mechanical behavior of dermis at this loading rate.

**Figure 2:**
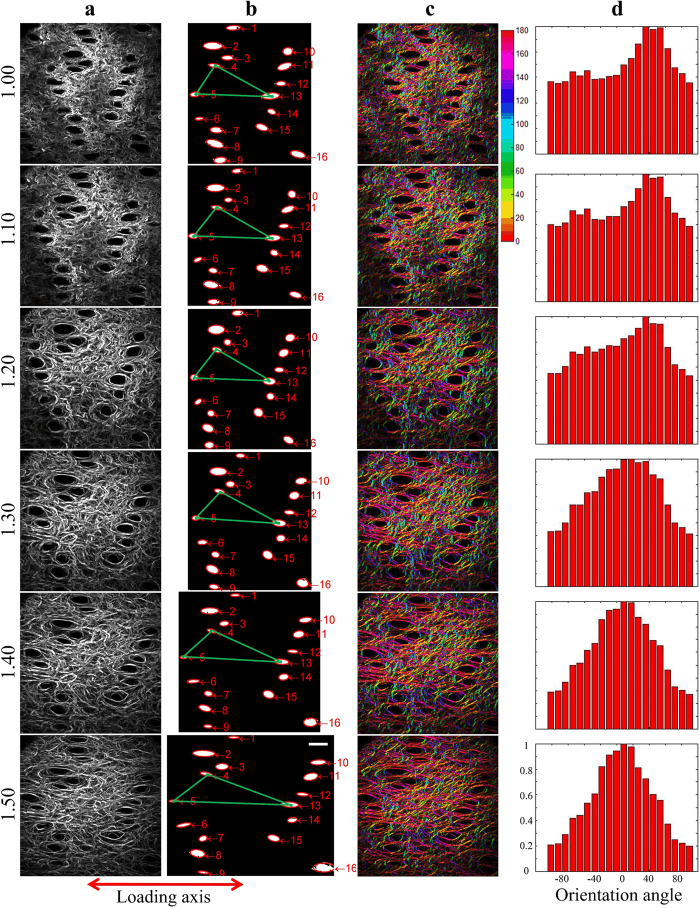
SHG imaging and processing of *ex vivo* stretched skin sample from *K14-COL5A1* mouse. (**a**) Raw SHG image of the dermis as a function of increased skin global deformation (top to bottom). Image size: 480 × 480 μm^2^. (**b**) Hair follicle segmentation revealing skin local deformation. Scale bar: 50 μm. (**c**) Processed SHG image (structuring element of 21 pixels) highlighting fiber orientations in every pixel with color code. Image size: 480 × 480 μm^2^. (**d**) Orientation histograms showing collagen increasing alignment along the traction direction.

SHG images revealed a meshwork of collagen fibrillar structures in the papillary dermis, which appeared increasingly thicker as we reached the reticular dermis, with no clear frontier between the two layers (see Supplementary Movie 1). Dark elliptical regions with no SHG signal corresponded to the location of hair follicles and the 2PEF signal inside these structures was characteristic for remaining keratin of hair shafts (see Supplementary Fig. S2). Upon traction, the collagen meshwork increasingly aligned along the traction direction and the follicle dark regions became more elliptical (Fig. 2a), as expected^24^,^49^.

### Non-invasive local deformation measurements

We first processed the SHG images to measure the local deformation tensor and verified whether it was equal to the global stretch ratio applied to the tissue. This issue was mandatory to compare the local data with the macroscopic ones since the skin tissue may exhibit an inhomogeneous response or slip slightly within the jaws. We therefore used the hair follicles as endogenous tags and measured the deformation of their network before and after stretching (see Material and Methods and Supplementary Fig. S3). To that end, appropriate image processing was developed to identify and label the same hair follicles in a region of interest (ROI) common to all images and to calculate the three independent components of the local deformation tensor using Delaunay triangulation (see Fig. 2b): the stretch ratios in the x and y directions, λ_xx_ and λ_yy_, and the sliding angle ω. This method cannot follow motions in the z direction (perpendicular to the skin surface), since the hair follicles extend through the whole thickness of the skin, thus the deformation tensor measured here is the in-plane one.

**Figure 3:**
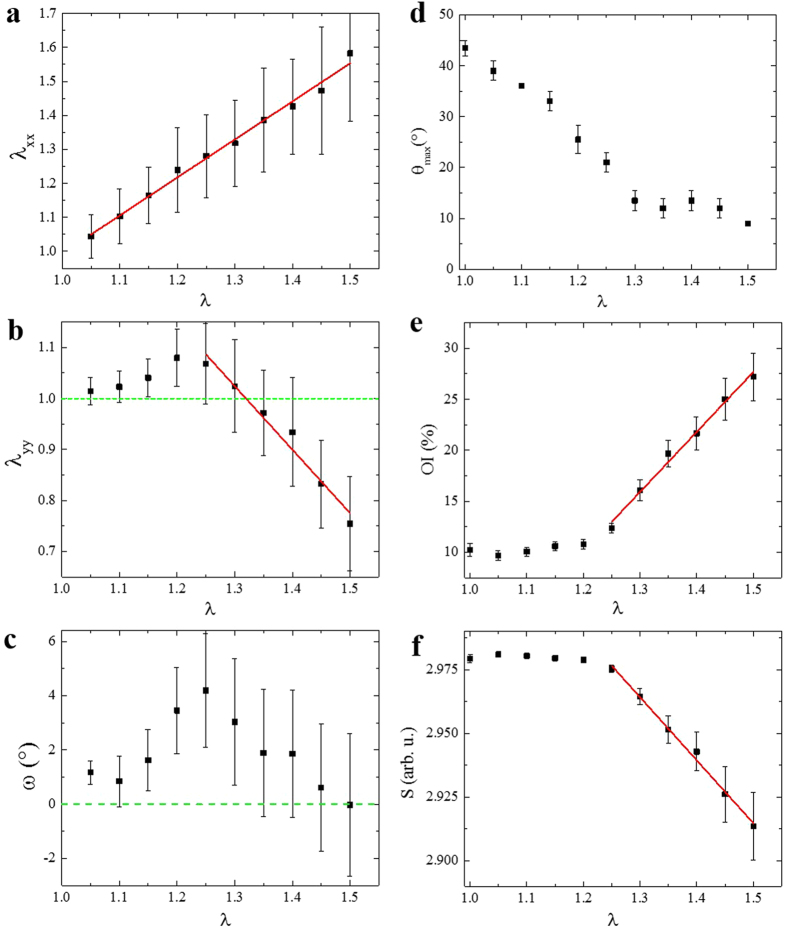
Local deformation and microstructural remodeling of skin sample from *K14-COL5A1* mouse (same mouse as Fig. 2). (**a**) λ_xx_, (**b**) λ_yy_ and (**c**) ω components of the local deformation tensor measured from hair follicle segmentation in the SHG image, x corresponding to the traction direction. Data points correspond to the average over the SHG cropped ROI (~300 × 300 μm^2^), with error bars calculated as the standard deviation. Solid red lines are linear fitting. The deformation along the traction direction λ_xx_ varies linearly as a function of the global stretch ratio, with a slope equal to unity, which shows that the global deformation is fully transmitted at the local scale. (**d–f**) Collagen reorganization probed by SHG imaging; (**d**) Main orientation angle θ_max_ of collagen fibers; (**e**) Orientation index (OI) of the collagen fibers; (**f**) Statistical entropy (S) of the orientation distribution. Data points correspond to the average over 6 consecutive images (10 μm) with error bars calculated as the standard error of the mean. Solid red lines are linear fitting. The collagen fibers align increasingly along the traction direction (corresponding to angle 0°). Note that λ_yy_, entropy and OI exhibit a bimodal behavior with slope switching at the same global stretch value (~1.25).

Figure 3a–c display the average value of these tensor components in the SHG image as a function of the global stretch ratio λ applied to the tissue for the same sample as in Fig. 2. The tensor component along the traction direction, λ_xx_, exhibited a linear behavior with a slope A_xx_ ≈ 1 and a slope intercept λ_xx,1_ ≈ 1. It proved that there was no sliding of the sample between the jaws and showed that the sample was homogeneous at the ROI scale (~300 × 300 μm^2^). The homogeneous values of the deformation maps (Supplementary Fig. S3), except few misplaced points, indicated that the skin was likely to be homogeneous at the scale of few tenth of micrometers.

The behavior along the y-direction, transverse to the traction, was more complex. It first slightly increased, which corresponded to a surface increase and would be associated to a negative Poisson’s ratio in linear materials. This peculiar behavior was likely due to water absorption at the beginning of the stretching^7^, although structural effects may also explain it (for example heterogeneous behavior along the skin thickness). Then it exhibited a linear contraction with a slope A_yy_ ≈ −1, for higher stretch ratios.

Finally, there was no observable shear at the scale of the ROI, the average sliding angle remaining small (<5°) throughout the experiment (Fig. 3c).

These results were highly reproducible for all the analyzed skin samples, which showed A_xx_ values close to 1 with a good fitting quality (R^2^ parameter), and exhibited a bimodal behavior for λ_yy_. It showed the reliability of mechanical measurements for both *WT* and transgenic mice (see Supplementary Tables S1 and S2).

### Microscopic scale collagen reorganization upon stretching

In parallel, collagen reorganization was quantified by extracting collagen orientation from SHG images at every deformation step. To that end, we used morphological filtering based on the surrounding pixels to create a colored coded orientation map of the fiber local orientation in every pixel (Fig. 2c and Supplementary Fig. S4). A normalized histogram of the fibers distribution in the ROI was then calculated (Fig. 2d). The initial histogram shows usually two wide peaks, or one wide peak with a shoulder, which seems to indicate pretension lines in murine skin similar to Langer’s lines in Humans^54^. These orientation histograms clearly showed that the collagen network rearranged during the traction assay from a more or less disorganized state (Fig. 2d, stretch ratio 1.0 – initial configuration), to a peaked distribution (Fig. 2d, stretch ratio 1.5) where the majority of fibers are parallel to the traction direction (0°).

To quantify this effect, we computed three parameters as previously described^55^: the main orientation angle (θ_max_), the orientation index in the main orientation (OI), which is maximal (100%) for perfectly aligned fibers and zero for disordered tissue, and the statistical entropy (S). Figure 3d–f display these parameters as a function of the global stretch ratio for the same skin sample as in Fig. 2. As expected, the main orientation angle tended to 0° (Fig. 3d). Moreover, the OI increased with the global stretch ratio, which indicated that more and more fibers were aligned in the main direction (Fig. 3e). Accordingly, the entropy decreased, showing that the tissue was globally more organized (Fig. 3f). Interestingly, the reorganization of the collagen fibers in the tissue appeared as a bimodal process. First the main orientation went to 0° with only a slight increase of OI and a slight decrease of entropy. Then θ_max_ was stable and the tissue better organized locally as indicated by the strong, almost linear, increase in OI and decrease in entropy.

**Figure 4:**
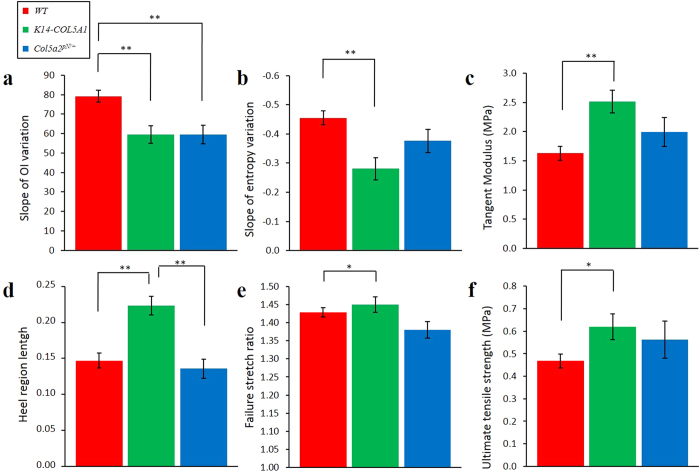
Multiscale biomechanical response of *WT*, *K14-COL5A1*, and *Col5a2*^*pN/*+^ murine skin. (**a**) Slope of OI variation upon stretching of skin samples from *WT*, *K14-COL5A1*, and *Col5a2*^*pN/*+^ mice; (**b**) Same with entropy; (**c**) Same with tangent modulus; (**d**) Same with heel region length; (**e**) Same with failure stretch ratio; (**f**) Same with ultimate tensile strength. Error bars corresponds to SEM. *(resp. **) indicates p < 0.05 (resp p < 0.01). Averaged values are calculated over all samples.

To quantify this local remodeling, we computed the slopes of the OI increase and of the entropy decrease using linear fitting for all the mice samples (Supplementary Tables S3 and S4). Averaged values for WT, *K14-COL5A1* and *Col5a2*^*pN/*+^ mice are displayed in Fig. 4a,b (see also Supplementary Fig. S5 for averaged values only over samples with both mechanical and SHG analysis). We observed statistically different results for WT and transgenic mice, both for OI and entropy. No difference was observed between *K14-COL5A1* and *Col5a2*^*pN/*+^ mice.

### Mechanical response of *WT*, *K14-COL5A1* and *Col5a2* ^*pN/*+^ skin

**Figure 5:**
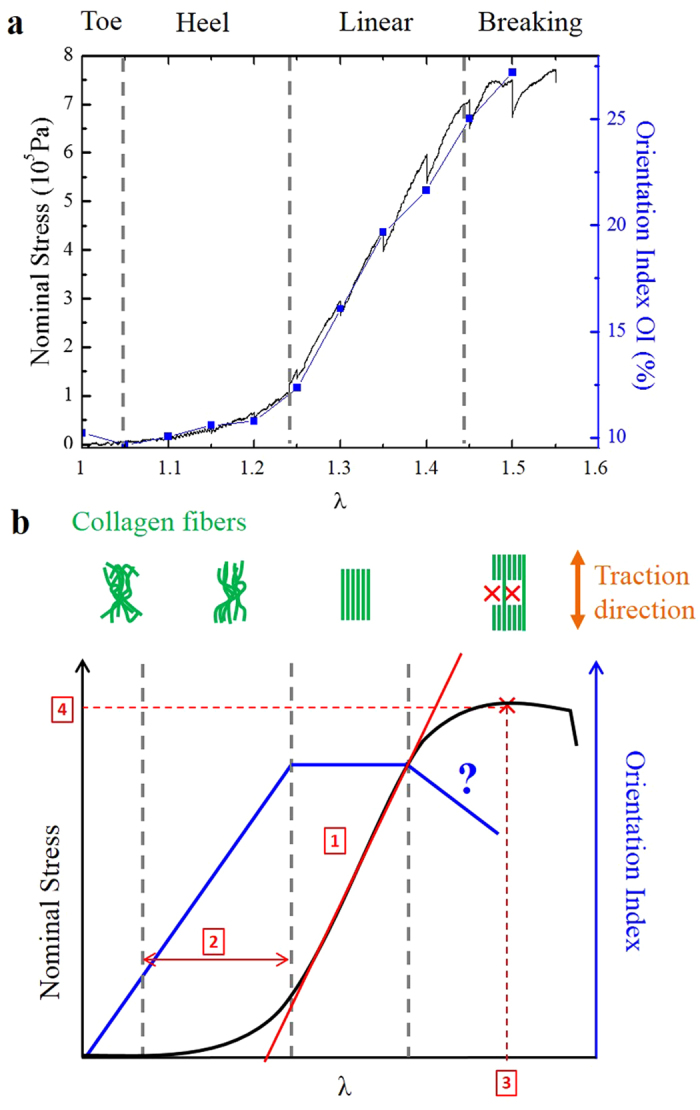
Multiscale biomechanical response of murine skin as compared to model tissue. Nominal stress (black) and Orientation Index (blue) variations as a function of the global stretch applied to skin sample from the same *K14-COL5A1* mouse as in Figs 2 and 3 (**a**) and to a model connective tissue (**b**). Both nominal stress and OI exhibit a linear dependence with strain in the same region. Four parameters characterize the mechanical response, as seen in (**b**), the tangent modulus (1), the heel region length (2), the failure stretch ratio (3) and the ultimate tensile strength (4). The OI behavior in (**b**) is extrapolated from the classical interpretation of the microstructural origin of the stress/stretch curve.

Mechanical macroscopic response was obtained from the force (F) and the initial sample section (S_0_) by calculating the nominal stress (F/S_0_); stretch ratio was quantified using the global imposed stretch ratio λ between the jaws. Figure 5 displays the stress/stretch curve obtained for the same murine skin sample as in Figs 2 and 3: it is similar to the typical stress/stretch curve (Fig. 5b) expected for skin and other collagen-rich tissues^8^. As depicted in Fig. 5b, it consisted of a toe region with no significant force below λ = 1.1, followed by a heel with increasing stiffness (slope of the stress/stretch curve) from λ = 1.1 to 1.25. Then the skin exhibited a linear behavior with constant stiffness (λ = 1.25 to 1.45). Finally over λ = 1.5 the stiffness decreased and the force saturated which indicated that the skin was breaking.

Four parameters were computed to quantify the mechanical response of each skin sample (Fig. 5b, in red): the tangent modulus, the heel region length, the failure stretch ratio and the ultimate tensile strength. The tangent modulus was obtained by measuring the slope of the linear part, approximately 1.2 MPa for the skin sample in Fig. 5a. The results for all skin samples are depicted in Supplementary Tables S5 and S6, and the averaged values for WT, *K14-COL5A1* and *Col5a2*^*pN/*+^ mice are displayed in Fig. 4c–f. Tangent modulus of WT and *K14-COL5A1* murine skin samples were statistically different (p < 0.01), but not those of WT and *Col5a2*^*pN/*+^ mice. The same statistical observation applied for failure stretch ratio and ultimate tensile strength (p < 0.05), and for the heel region length (p < 0.01). For the latter parameter only, *K14-COL5A1* and *Col5a2*^*pN/*+^ skin samples exhibited also significant differences (p < 0.01).

These different mechanical responses may be ascribed to a difference in the skin porosity, we thus quantified the hair density, the averaged surface of hair follicles and the resulting relative surface porosity of all samples. No significant difference in hair density or in relative surface porosity were observed between WT and *K14-COL5A1* or *Col5a2*^*pN/*+^ skins (Supplementary Fig. S6). A significant difference was observed between *K14-COL5A1* and *Col5a2*^*pN/*+^ skins in terms of averaged hair follicles surface and thus in relative porosity. However, this 2% change in relative porosity was too small to explain the observed mechanical differences. No significant thickness difference was observed between *WT*, *K14-COL5A1* and *Col5a2*^*pN/*+^ skins.

Finally, we plotted the OI variation with stretch ratio in the same graph as the stress/stretch curve (Fig. 5a). We observed that both parameters exhibited the same behavior, although OI quantifies collagen remodeling at microscopic scale and stress measures the mechanical response at macroscopic scale. This result was consistent for all the samples.

## Discussion

Advanced investigations of skin biomechanics have been impeded so far by technical limitations since it requires simultaneous measurements of the mechanical response at macroscopic scale and of the tissue reorganization at microscopic scale, which is highly challenging in such a complex tissue. To overcome this bottleneck, we developed an experimental set-up combining a traction device with a SHG microscope and we imaged *in situ* the collagen network in stretched murine skin up to skin rupture. All these measurements were performed in *ex vivo* murine skin with well-preserved structures by designing appropriate protocols.

We also thoroughly verified the reliability of our mechanical measurements to eliminate possible artefacts that could affect measurements in complex biological tissues. First, the dark area around the hair shafts in the SHG images was used as endogenous tags to measure the local stretching in a non-invasive way. The quantitative agreement between measurements of this local stretch ratio and of the macroscopic stretch ratio proved that the global deformation was fully transmitted to the skin tissue without any sliding and that the sample response was homogeneous at the local scale (see Fig. 3 and Supplementary Tables S1 and S2). Secondly, we verified using the SHG images that the mechanical response was not affected by changes in skin thickness or surface porosity between WT and genetically-modified skins. Finally, we implemented an automated image processing protocol to quantify collagen reorganization in an efficient way.

Our multiscale approach thus enables robust characterization of skin biomechanics as shown by the very good reproducibility of all our measurements (Fig. 4 and Supplementary Tables S1 to S6). It provides unique quantitative information about collagen reorganization at micrometer scale and stress at macroscopic scale, in a native environment, as a function of controlled stretch measured at a scale of few hundredth micrometers (the resolution being limited by the distance between hair follicles). Both papillary and reticular dermis are probed by this method, while continuous change of skin structure along its depth, as visualized by SHG, showed that there was no abrupt frontier between reticular and papillary dermis, but a progressive variation of matrix architecture.

Our measured stress/stretch relationship of the skin is similar to previously reported^7^,^8^,^9^: a heel region followed by a linear part before breaking. Our approach distinctively allows a quantitative challenge of the microstructural origin of the stress/stretch curve, which classically associates the heel region to an alignment of the fibers and the linear one to the stretching of the fibers. Extrapolating this interpretation in term of fibers orientation, we should observe an increase of the OI in the heel region, followed by a plateau (or a reduced increase) in the linear region, and a decrease after breaking (Fig. 5b, blue straight line). Our OI measurements did not sustain this interpretation. The evolution of the OI with the stretch ratio (Fig. 5a) indicates that the heel region is associated to only a very partial alignment of the fibers^49^ and that the linear region corresponds to the maximal alignment of the collagen fibers, as also observed on the orientation histograms (Fig. 2d). Similar results are obtained for the entropy, as expected for uniaxial traction. The increase of the stress in the linear part might thus be associated to the increase of the fraction of fibers aligned in the direction of traction instead of the stretching of the fibers. In that case, each aligned fiber would provide a given stress to the tissue, whatever the stretch ratio is. Such behavior remains a plastic deformation of the fibers, and could originate from the sliding of fibers as reported in tendon^22^,^23^. It might be that sliding occurs above a critical value of load, associated with breaking of cross-bridges and that each stretched fiber contributes only by this critical load to the total stress applied to the sample.

This multiscale approach was instrumental to analyze the skin mechanical properties of two different genetically-modified murine mutant lines, in which collagen V expression has been modified: the transgenic *K14-COL5A1*^50^ and the knock-in *Col5a2*^*pN/*+^ mice^4^,^5^. Although defects in skin biomechanics represent the hallmark of cEDS, the biomechanics of the *Col5a2*^*pN/*+^ skin have not been investigated. *K14-COL5A1* mouse line was generated to analyze the role of the homotrimer [α_1_(V)]_3_, a presumed minor molecular species in skin. Overexpression of this collagen V subtype in *K14-COL5A1* skin provoked ultrastructural changes at the epidermis-dermis interface that resulted in changes in the biomechanical properties of the transgenic skin^50^.

We found that the reorganization of collagen fibers was higher in WT than for the two mutants while they both displayed identical responses. While the mechanical response was modified in *K14-COL5A1* mice with respect to WT, the *Col5a2*^*pN/*+^ mice were statistically identical to WT from a mechanical point of view.

The stress/stretch relationship for *K14-COL5A1*^*WT*^ and the transgenic *K14-COL5A1*^50^ previously showed different results, with much closer tangent moduli. However, many experimental differences may explain this disagreement. First, the loading rate was very different (0.17% s^−1^ versus 0.01% s^−1^ here). Second, the orientation of the skin was not reported, which could modify the mechanical response. Finally, the samples were fully immerged, which could lead to a not physiological, major, absorption of water.

The reorganization of the fibers is characterized by two quantitative parameters: the size of the heel region and the slope of the OI. As the stress appears to be proportional to the OI, the mechanical properties before rupture are characterized only by a single parameter; we consider here the tangent modulus. The change in collagen reorganization (OI and length of the heel region) for similar stretch ratios between WT and mutant mice implies that the alignment of the collagen fibers is at least partially controlled by the viscous properties of the skin. If it was a purely elastic process, as the deformations are the same for all mice, the motion of the fibers would be also the same, even if the stresses are different. Our data thus show that the fibers move independently from the skin deformation, indicating that inner relaxation processes of the skin are involved. This is in agreement with the fact that each fiber carries only a given stress: the sliding between fibers is also associated with a transmission of the stress between fibers through the non-fibrillar matrix.

However, the length of the heel region is different between the two genetically-modified mice while the slope of the OI remains the same. This indicates that these two parameters correspond to different physical processes. In the heel region, we observed only a small alignment of the fibers, but a change of the main orientation of the fibers; it corresponded also to the region of expansion in the transverse directions. Thus, we consider here that the heel region is associated with a reorganization of the non-fibrillar matrix at the tissue scale, associated with water exchange; only a small fraction of fibers are then involved in the mechanical behavior. The slope of the OI is associated to the behavior in the linear region of the stress/stretch curve. A fast increase of the OI is thus related to an easier alignment of the fibers, with less or no water exchange. It can then be associated mainly to a reorganization of the fibers network, the non-fibrillar matrix playing likely a smaller role except as bridging fibers.

Our approach allows connecting observations of the ECM organization to the mechanical properties of the tissue making possible to distinguish phenotypes that are undistinguishable at the tissue scale. *Col5a2*^*pN/*+^ mice was shown to mimic the hyperextensibility of cEDS skin but exhibited mechanical properties indistinguishable of the ones of WT. This result was by itself a surprise. The cEDS phenotype is probably milder in heterozygous compared to homozygous mice. However, cEDS is an autosomal dominant disorder. Patients are thus heterozygous for mutations in either *COL5A1* or *COL5A2* although they have abnormally stretchy skin. It is also worth noticing that the initial organization of the fibers cannot be distinguished between *Col5a2*^*pN/*+^ and WT mice. However, our multi-scale approach allows us to detect a difference in the reorganization of the collagen fibers between *Col5a2*^*pN/*+^ and WT mice. Thus, even on complex modifications of the skin microstructure (disorganization of the extracellular matrix^4^,^5^,^6^, increase in variability in collagen I/V fibers size), our setup is able to distinguish different phenotypes, even for undistinguishable mice at the full body level.

Finally, our experimental setup also provides insight on the skin microstructural modifications. In *K14-COL5A1* skin, a decrease of the reorganization, with an increase of the length of the heel region and a decrease of the slope of the OI was observed. Mechanically, we observed also an increase of the tangent modulus. The lower reorganization and higher tangent modulus in the linear part implies that the fibers are harder to rotate but are able to carry a higher load. This is well explained by the addition of a network of very small fibers, not visible with SHG, that cannot detect objects smaller than 30nm^40^. Such explanation is in agreement with TEM observations of the extracellular matrix modifications in *K14-COL5A1* skin that revealed the presence of very small collagen V fibers in papillary dermis. This network entangles the collagen fibers, preventing their rotations, while increasing the friction between fibers, and thus increasing the stress per fiber. The increase in the non-fibrillar matrix viscosity associated with the addition of this network leads also to lower water exchange and lower reorganization, explaining a longer heel region.

In conclusion, the approach developed here allows a careful and robust analysis of the changes in microstructural organization of the dermis as a response to mechanical loading. It shows a strong correlation between the macroscopic force and the fraction of aligned fibers, which might indicate that each fiber provides only a given force. Moreover, application of this approach to transgenic mice with modified expression of collagen V gives new insights into the role of collagen V molecular species in dermis. This work also provides an efficient tool to investigate the biomechanics of collagen-rich tissues in normal and pathological context and to guide tissue engineering with appropriate biomechanical responses.

## Materials and Methods

### Mice models

#### Genotypes

Two different mice models were used in this study. The first one is the transgenic *K14-COL5A1* mouse line overexpressing the human proα1(V) chain in the epidermis under the control of the K14 promoter^56^ that was created and characterized previously^50^. The second one is the *Col5a2*^*pN/*+^ line (deletion of exon 6 in *Col5a2* gene) which is a mouse model for cEDS^4^,^5^.

All animal experiments were performed under animal care procedures and conducted in accordance with the guidelines set by the European Community Council Directives (86/609/EEC). All experimental procedures were approved by the Direction of the Veterinary Service of Rhone Department (DDSV, Lyon, France). 52 mice were used: 9 *K14-COL5A1*, 18 *Col5a2*^*pN/*+^ and 25 *WT* (obtained from the same litters) (see Supplementary Tables S1–S6). Wild-type specimens referred as *WT* hereafter were obtained from 129sv mice, or by crossing transgenic mice *K14-COL5A1 (K14-COL5A1*^*WT*^) or *Col5a2*^*pN/*+^ heterozygous mice (*Col5a2*^+ */*+^).

#### Skin samples

Mice were sacrificed at one month by cervical dislocation and the skin of the back was shaved with an electric shaver. Depilatory cream was applied during 15 minutes and hairs were removed with a scraper. The skin of the back was collected and the right foreleg was spotted with black ink to identify the head-tail axis. In order to separate epidermis from dermis and facilitate dermis imaging (preventing light absorption by melanin in the epidermal cells and reducing the skin thickness to image deeper into the dermis), skin was then incubated 30 minutes at room temperature, with 3.8% ammonium thiocyanate^57^. Skin samples were stored in culture medium (Dulbecco’s Modified Eagle’s Medium, Sigma-Aldrich) without phenol red, supplemented with 50 μg/mL penicillin/streptomycin (Sigma) at 6°C and used within five days for the biomechanical experiments. Ear biopsies were systematically collected for genotyping analysis. Genotyping of *Col5a2*^*pN/*+^ and *K14-COL5A1* mice was then performed by PCR as described^4^,^50^. Measurements and mice genotyping were performed in a double-blind fashion.

#### Histological analysis

See Supplementary Material.

### Combination of multiphoton imaging and mechanical assays

#### Traction device

Mechanical assays were performed using a custom-built uniaxial traction device, inserted in place of the microscope stage. This device is composed of two motors (drl42pa2g-04; Oriental Motor, Tokyo, Japan) and two force sensors (LPM200, 2lb, Futek, USA) on each side of the sample. Displacement of the motors was imposed and the resulting force recorded every second. The traction was symmetric to enable semi-continuous imaging of the same region of interest (ROI) (Fig. 1a,b).

Samples were cut into a dog-bone shape to ensure homogeneous uniaxial tensile load in the central testing portion (Supplementary Fig. S2). The traction was applied in the head-tail direction, with the papillary dermis up facing the objective lens (Supplementary Fig. S2). A drop of immersion gel (Lacrygel, Europhta) ensured optical contact with the objective lens and prevented skin dehydration during experiments.

#### Multiphoton microscopy

Multiphoton imaging was performed using a custom-built laser scanning microscope as previously described^12^,^20^ (Fig. 1a and Supplementary Material). Signals were collected using 100 kHz pixel rate with 0.5 μm pixel size, 2 μm axial steps and 30 mW typical laser power at focus. Image stacks were typically 480 × 480 × 50 μm^3^ (5 minutes recording time). No degradation of the skin samples was usually observed under these conditions.

#### Mechanical assays under multiphoton microscope

The reference dimensions of each sample (length l_0_, width w_0_ and thickness e_0_) were quantified using a digital caliper (see Supplementary Material for definition of reference position). Typical size of the sample was 20 × 8 × 1 mm^3^, with about 0.1 mm accuracy (see Supplementary Fig. S2).

The tensile test was then performed at a fixed strain rate, chosen deliberately slow at 10^−4^ s^−1^ (typically 2 μm.s^−1^) to enable monitoring of the same ROI. SHG imaging was not possible during traction because of skin movements during the ≈5 minutes of imaging. The loading path was thus chosen incremental: we stretched the sample by a step of 0.05 stretch ratio, recorded a SHG/2PEF images z-stack while keeping the deformation constant, and carried on with stretching the sample. We checked that we always imaged the same ROI by looking at characteristic patterns from hair follicles. When necessary, we slightly adjusted the lateral and axial positions of the skin sample by moving the whole traction device by means of micrometer stages. We carried on this way until breakage of the sample, usually around a stretch ratio of 1.5, after approximately 3 hours experiment.

#### Mechanical assays without multiphoton imaging

See Supplementary Material.

### Mechanical data processing

The motor displacement and resulting force were measured continuously during the whole experiments (Fig. 1c). The global stretch ratio was obtained as:


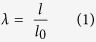


where *l* is the length between the jaws and *l*_*0*_ the reference length. Note that because of the uncertainty in the reference length, the absolute stretch is not precisely defined; nevertheless, the differences in stretch ratio are accurately determined.

The nominal stress was obtained as the measured force divided by the initial skin section (w_0_ × e_0_). Also because of the uncertainty in the skin section measurements, the absolute stress is not precisely measured, while the variations of stress are accurately determined.

To quantify the mechanical properties of the sample, we used four parameters (Fig. 5 and Supplementary Tables S5–S6). First, the tangent modulus characterized the stiffness of the sample. It corresponds to the slope of the linear part of the stress/stretch curve. The linear part was defined manually for each sample. The fit was carried out on the linear part leaving out the pauses made for imaging, from the beginning of the pause until stress reaches again the value it had before pausing.

Second, the length of the heel region corresponds to the non-linear response of the material, classically attributed to the alignment of the collagen fibers. The noise was initially measured for each test at very small stretch ratio (a few percent). The start of the heel region was defined by the first point at which the stress stayed over twice the noise value for at least 10 seconds. We defined the end of the heel region as the point at which the stress got close enough to the linear part regression, *i.e*. when the difference became inferior to twice the noise.

Third, we quantified the resistance to rupture with the failure stretch ratio and ultimate tensile strength. We considered the maximum stress to be the “ultimate tensile strength”, and the stretch ratio for which that stress value was obtained the “failure stretch ratio”.

### SHG image processing

SHG images showed fibrillar structures corresponding to collagen, interrupted by round structures with no SHG signal and corresponding to the hair follicles (Supplementary Fig. S2). Three types of information were then obtained: the local stretch in the ROI (~300 × 300 μm^2^), the skin porosity, and the local organization of fibrillar collagen. To that end, specific image processing was developed using a custom-written MATLAB script (The MathWorks, Natick, USA).

#### Local stretch

The centers of all follicles were identified and labelled (see Supplementary Material) to define a network at each deformation step and calculate the local deformation tensor. To that end, we performed a Delaunay triangulation and calculated for each triangle (Supplementary Fig. S3) the deformation tensor relative to the initial triangle in the non-stretched state. This deformation tensor exhibited three independent components λ_xx_, λ_yy_ and the sliding angle ω representing respectively the stretch ratios in x and y direction and the shear in the ROI. Thus, at each deformation step, we obtained three maps of deformation (Supplementary Fig. S3). Finally, the averages of each tensor component provided a measure of the local deformation in the ROI, which was compared to the global stretch ratio applied to the skin sample (Fig. 3a–c and Supplementary Tables S3 and S4).

#### *Skin porosity*

##### Fiber orientation

Independently, we extracted fibers orientations from SHG image stacks using morphological filtering by a rotating linear structuring element as previously reported^40^ (see Supplementary Material).

To quantify the degree of organization of the tissue, we used three parameters as previously described^55^: (i) the main orientation θ_max_ in the ROI, which corresponds to the maximum of the orientation histogram, (ii) the orientation index (OI) in the main orientation:


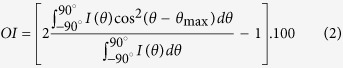


and (iii) the statistical entropy:


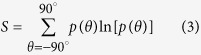


where


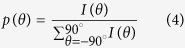


is the probability to find a fiber in the direction θ which is directly given by the orientation histogram.

### Statistics

All the skin samples were included in the statistical analysis, unless experimental data were not available for the following reasons: technical problem in the mechanical data acquisition (sensor error…) or in SHG image recording (air bubbles…) or too short linear part in the OI or S variation. Error bars correspond to Standard Error of the Mean. Statistical tests were performed with R (R development core team, R foundation for statistical computing). We verified both the normality of the distribution and the equality of variances using respectively the Shapiro-Wilk test and the Fisher test. Therefore, the significance of the mean differences was determined using sided two-sample t-test. All tests were performed using an alpha level of 5%.

## Additional Information

**How to cite this article**: Bancelin, S. *et al*. *Ex vivo* multiscale quantitation of skin biomechanics in wild-type and genetically-modified mice using multiphoton microscopy. *Sci. Rep*. **5**, 17635; doi: 10.1038/srep17635 (2015).

## Supplementary Material

Supplementary Information

Supplementary Movie S1
