# Supplementary material for: Ex vivo multiscale quantitation of skin biomechanics in wild-type and genetically-modified mice using multiphoton microscopy
Source: Sci Rep. 2015 Dec 3;5:17635. doi: 10.1038/srep17635 (PMC4668561; doi:10.1038/srep17635)
Supplement: Supplementary Information [file srep17635-s1.pdf]

Supplementary information

Ex vivo multiscale quantitation of skin biomechanics in wild-type and genetically-modified mice using multiphoton microscopy

Stéphane Bancelin[#(1)], Barbara Lynch[#(2)], Christelle Bonod-Bidaud[#(3)], Guillaume Ducourthial[(1)], Sotiris Psilodimitrakopoulos[(1)], Petr Dokladal[(4)], Jean-Marc Allain[(2)†], Marie-Claire Schanne-Klein[(1) †*], Florence Ruggiero[(3) †]

(1) Laboratory for Optics and Biosciences, Ecole Polytechnique, CNRS, INSERM U1182, 91128 Palaiseau Cedex, FRANCE

(2) Laboratory of Solid Mechanics, Ecole Polytechnique, CNRS, Mines ParisTech, 91128 Palaiseau Cedex, FRANCE

(3) Institut de Génomique Fonctionnelle de Lyon, ENS-Lyon, CNRS UMR 5242, Université Lyon 1, 46 Allée d'Italie, 69364 Lyon, cedex 07 France

(4) Centre for Mathematical Morphology, MINES ParisTech, PSL Research University, 35 rue St Honoré, 77300 Fontainebleau, France

# These authors contributed equally to this work.

[†] allain@lms.polytechnique.fr, marie-claire.schanne-klein@polytechnique.edu, florence.ruggiero@ens-lyon.fr

Supplementary Methods

Histological analysis:

For histology, total skin or skin without epidermis, were fixed in 4% PFA, embedded in paraffin, and 5 μm thick sections were performed. Deparaffined sections were then stained with the Masson's trichrome method or Herovici's staining (Novotec, Lyon, France).

Multiphoton microscopy

Excitation was provided by a femtosecond Ti-Sa laser tuned at 860 nm (Tsunami, Spectra-Physics). Samples were illuminated in an upright geometry using quasi-circularly polarized excitation at 860 nm (ellipticity 0.12) to enable homogeneous imaging of fibers whatever their orientation in the focal plane. SHG and 2PEF signals were recorded in backward direction using photon-counting photomultiplier tubes (P25PC, Electron Tubes) and appropriate dichroic mirror and spectral filters (FF01-680/SP, FF01-720/SP and FF01-427/10, Semrock for SHG and FF01-680/SP, FF01-720/SP and GG455, Semrock for 2PEF). High numerical aperture water immersion objective (20x, NA 0.95, Olympus) was used and typical lateral and axial resolutions of $0.4 \times 1.6\ \mu m^2$ were obtained near the sample surface.

Determination of reference position for mechanical measurements

Skin samples were attached to the traction device by use of metallic jaws and placed under the multiphoton microscope. The macroscopic folds due sample slenderness were unfolded to define the reference configuration. Making sure the force did not exceed the sensors noise (0.03 N), the metallic jaws were moved apart slowly (usually around 0.01 mm s^{-1}) while the sample was continuously imaged by SHG microscopy until no more vertical displacement could be observed. Considering the axial resolution within the dermis, vertical displacement was observed typically with 3 μm accuracy. This position was referred to as the initial position and used as reference. This position is not the first one at which a measurable force is recorded (which corresponds to the starting of the heel region): the region in-between is often called the toe-region.

Mechanical assays without multiphoton imaging:

Some mechanical assays were also performed without multiphoton imaging. In that case, the samples were cut into a dumbbell shape and then marked with dots of Indian ink on the papillary dermis side using a soft brush to create a pattern resistant to water. They were then attached to the traction device as described before. The reference configuration was determined by

stretching slowly until a small force was detected (usually 0.02 N) and traction was performed at 10^{-4} s^{-1} without stopping, until breakage of the sample. Constant hydration was obtained by spraying mineral water on the sample every 3 minutes. A camera was used to record a picture of the sample every second and Digital Image Correlation was performed using CorrelManuV software[1] to correct for possible slippage.

Follicle segmentation from the SHG images:

SHG images at approximately the same depth within the skin sample were cropped to obtain a ROI common to every deformation step that is displaying the same hair follicles. Size of this ROI increased in the stretching direction upon increasing deformation as exemplified in Fig. 2b. A median filter (2 pixels radius) was applied to reduce noise and the contrast was inversed (Supplementary Fig. S3). Circular opening was used to smooth the image and preserve the edge of the hair follicles. Then a threshold (≈250) was applied to the 8-bits images to create binary images and a shape detection was performed to identify and label every follicles in the ROI. Finally, the follicles were renumbered to obtain the same order at each stretch ratio (Fig. 2b).

Skin porosity:

The number of hair follicles and their size in the initial cropped image (zero stretch ratio) were measured from the segmented SHG images (see above) and the sample relative surface porosity was computed as the product of these two measures over the size of the initial image. These data obtained within a ROI of typically 300×300 μm^2 (cropped ROI) were then extrapolated at the scale of a millimeter square, by means of two parameters: initial number of hair per millimeter square (or hair density) and initial relative surface porosity.

Fiber orientation:

SHG images were filtered out using a mean filter (radius 3 pixels) and a morphological opening[2] was applied using a linear structuring element (Strel) of 21 pixels (9 µm) (Supplementary Fig. S4), in order to extract the fibers along the Strel direction. By rotating the Strel, we obtained a stack of images, each one corresponding to a Strel direction. The pixel by pixel maximum in the stack then provided an enhanced image of the collagen fibers in the sample and the direction in the stack realizing this maximum provided the fiber orientation. We thus obtained for each pixel of the SHG image the local orientation of the collagen fibers, which was color coded to obtain an orientation map with enhanced fibers (Supplementary Fig. S4 and Fig. 2c). We finally calculated normalized histograms $I(\theta)$ of the fibers orientation (Fig. 2d). The number of accessible angles depends on the length of the structuring element. A long Strel allows indeed for a fine angular discretization step but the length is upper-bounded by the curvature of the

fibers that a long Strel needs to fit in. Note that this method advantageously extract the orientation of fibrillar structures, while mitigating any artifact due to image edges[2].

Finally, the three orientation parameters (main angle, OI and entropy) were computed from 6 consecutive SHG images in the z-stack (10 μm thick) corresponding to the maximal signal density.

References:

1. Bornert M, Vales F, Gharbi H, & Nguyen Minh D. Multiscale full-field strain measurements for micromechanical investigations of the hydromechanical behaviour of clayey rocks. *Strain* **46**, 33-46 (2010).
2. J. Serra, *Image Analysis and Mathematical Morphology* (Academic Press, 1982).

Supplementary Video

Video Title: Multiphoton z-stack of the skin dermis of a Wild-Type mouse

Video caption: z-stack of combined SHG (green) and 2PEF (red) images of skin dermis. Images are recorded every 2 μm from the epidermis side and show continuous transition from the papillary to the reticular dermis.

Supplementary Figures

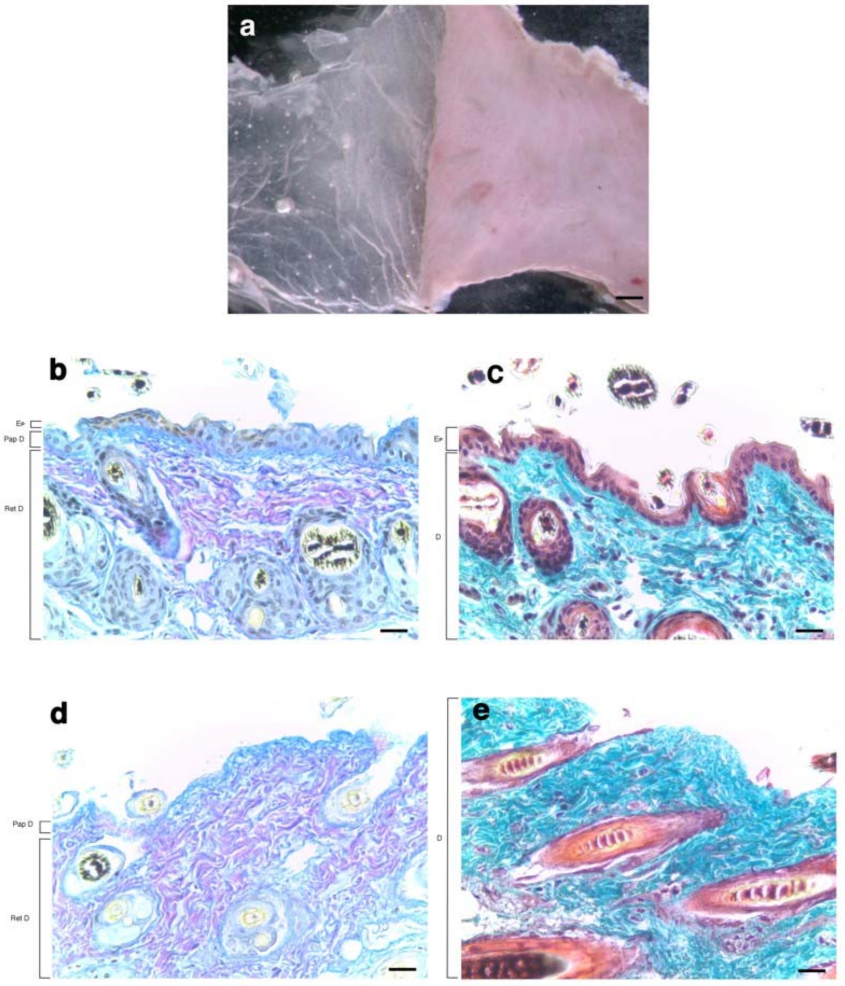

Supplementary Figure S1: Skin histology.

(**a**) Skin was dissected from one month Wild-Type (*WT*) mice. Depilated epidermis was separated from dermis with ammonium thiocyanate. Scale bar: 0.5 cm. Total skin (**b-c**) and dermis without epidermis (**d-e**) were stained with the Masson's trichrome (**c-e**) or Herovici's methods (**b-d**). Ep = epidermis; D = dermis. Scale bars: 20 µm.

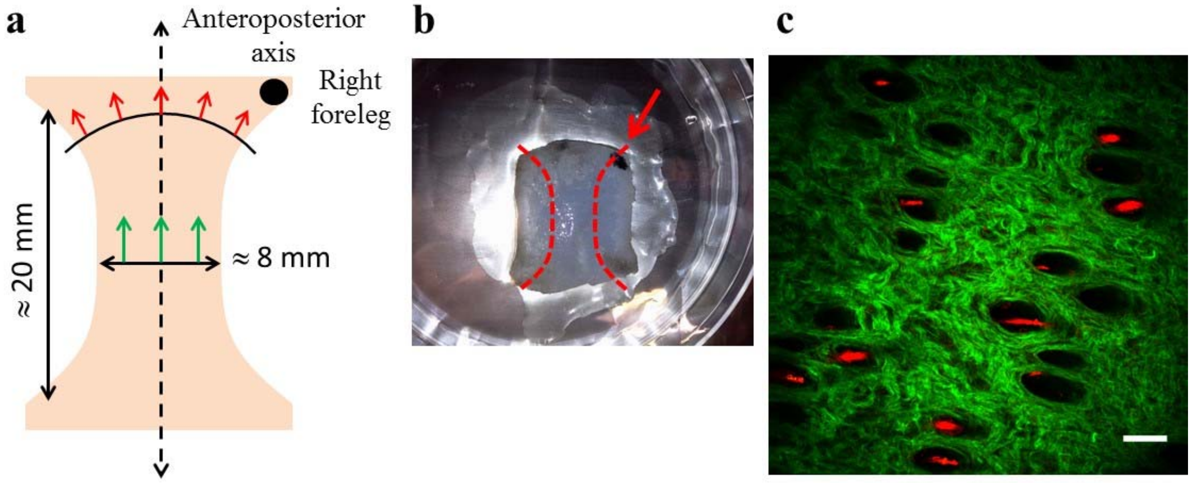

Supplementary Figure S2: Skin biopsy handling.

(**a**) Scheme and dimensions of the shaped skin biopsy along the anterio-posterior axis of the mouse back; (**b**) View of the skin biopsy before cutting; the black mark corresponds to the right foreleg; (**c**) multiphoton image of the skin reticular dermis. SHG (green) reveals fibrillar collagen, while 2PEF (red) reveals residues of hair follicles. Scale bar: 50 µm.

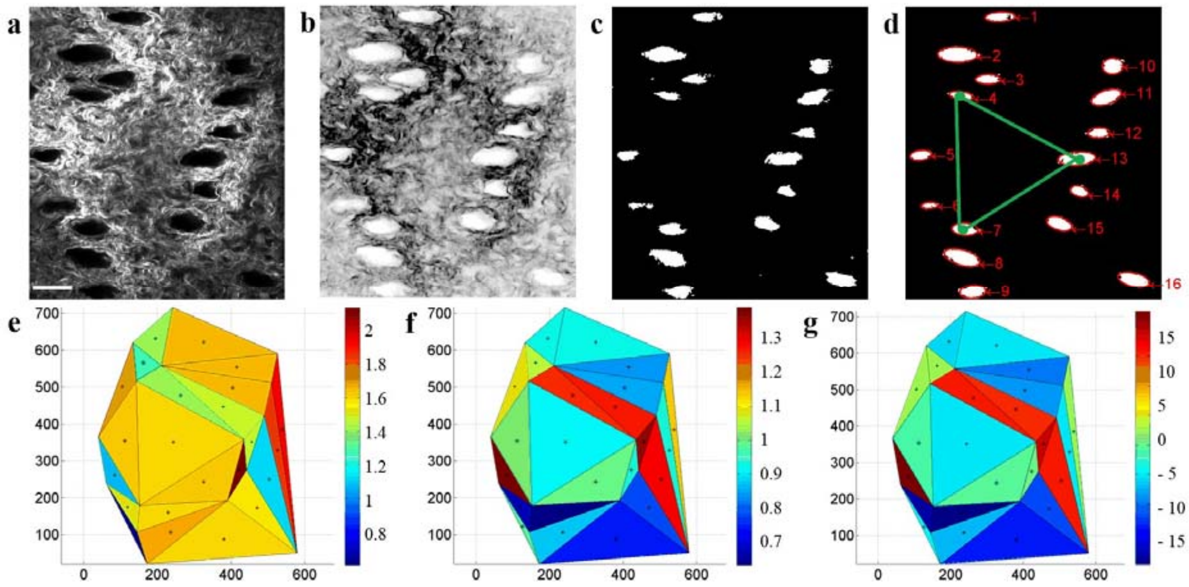

Supplementary Figure S3: Hair follicle segmentation in the SHG image for local deformation measurement.

(**a**) Raw and (**b**) enhanced SHG image of skin papillary dermis from *K14-COL5A1* mouse; (**c**) Binary SHG image after thresholding; (**d**) Position detection and numbering of hair follicles; Mapping of (**e**) λ_{xx}, (**f**) λ_{yy} and (**g**) ω tensorial components calculated from the deformation of the hair follicle network –see green triangle in (d) - compared to the initial network before stretching. Scale bar: 50 µm.

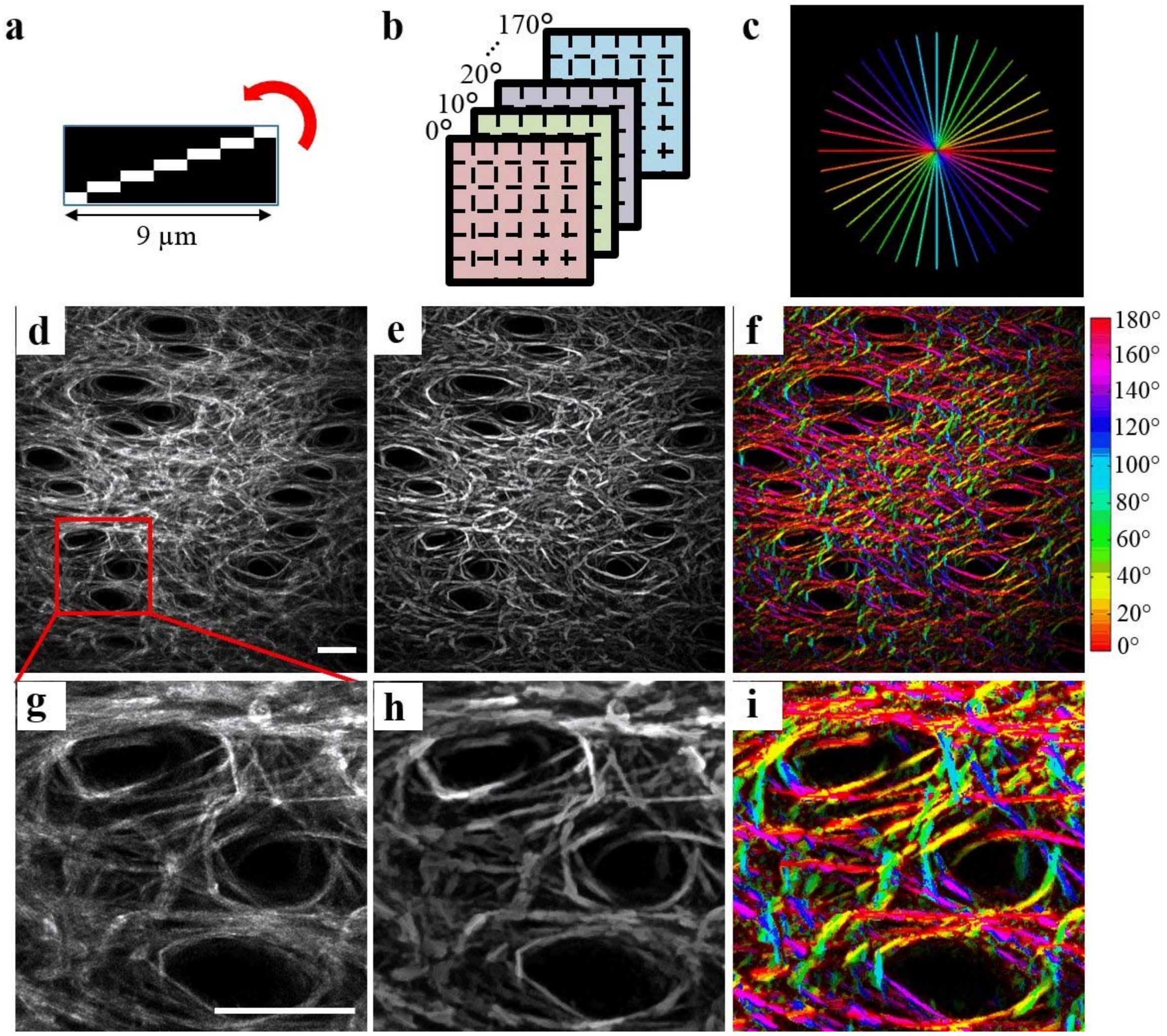

Supplementary Figure S4: Collagen fiber orientation measured from the SHG image.

(**a, b**) Rotating elementary structure (21 pixels long, angle 20°) used to filter out the SHG image along various orientations; (**c**) Orientation determination on a model image; (**d, g**) Raw and (**e, h**) enhanced SHG image of skin dermis from *K14-COL5A1* mouse; **(f, i)** Orientation mapping using the angular look-up table on the right. Scale bars: 50 μm.

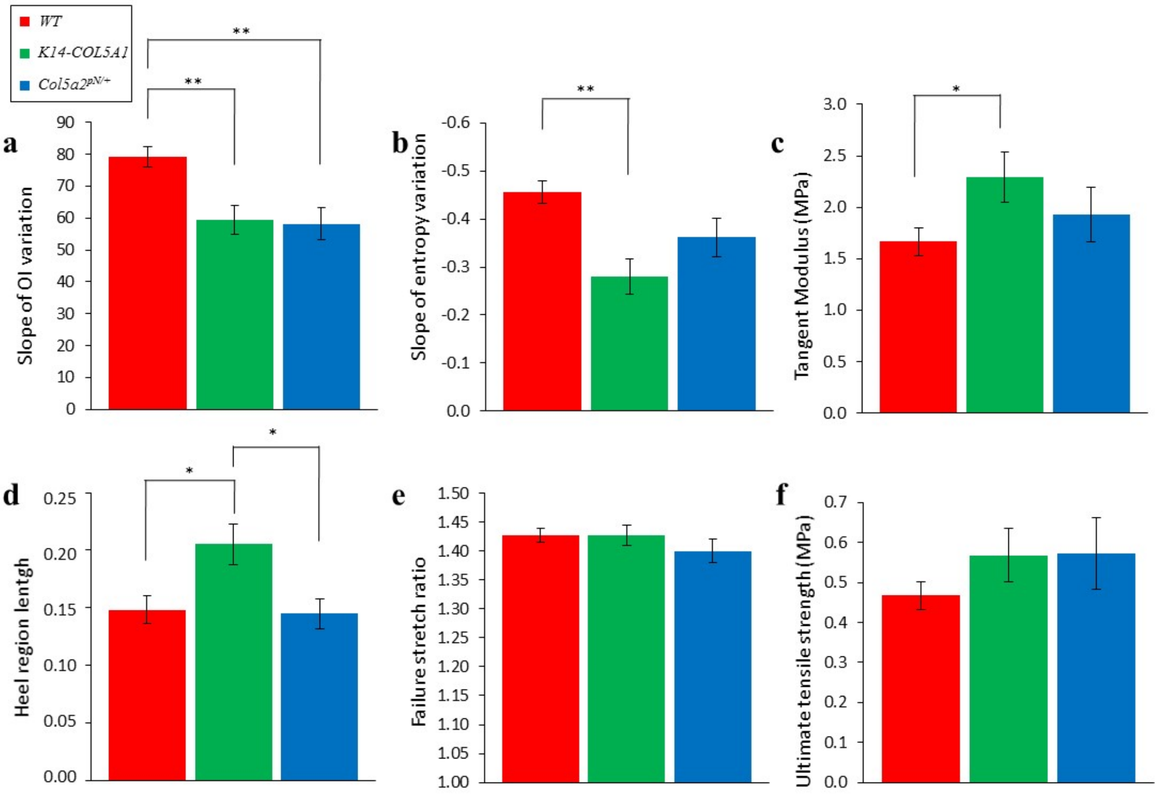

Supplementary Figure S5: Multiscale biomechanical response of *WT*, *K14-COL5A1*, and *Col5a2*$^{pN/+}$ murine skin.

Same as Figure 4 with averaged values only over samples with both mechanical and SHG analysis (**a**) Slope of OI variation upon stretching of skin samples from *WT*, *K14-COL5A1*, and *Col5a2*$^{pN/+}$ mice; (**b**) Same with entropy; (**c**) Same with tangent modulus; (**d**) Same with heel region length; (**e**) Same with ultimate stretch ratio; (**f**) Same with ultimate tensile strength. Error bars corresponds to SEM. * (resp. **) indicates $p<0.05$ (resp $p<0.01$).

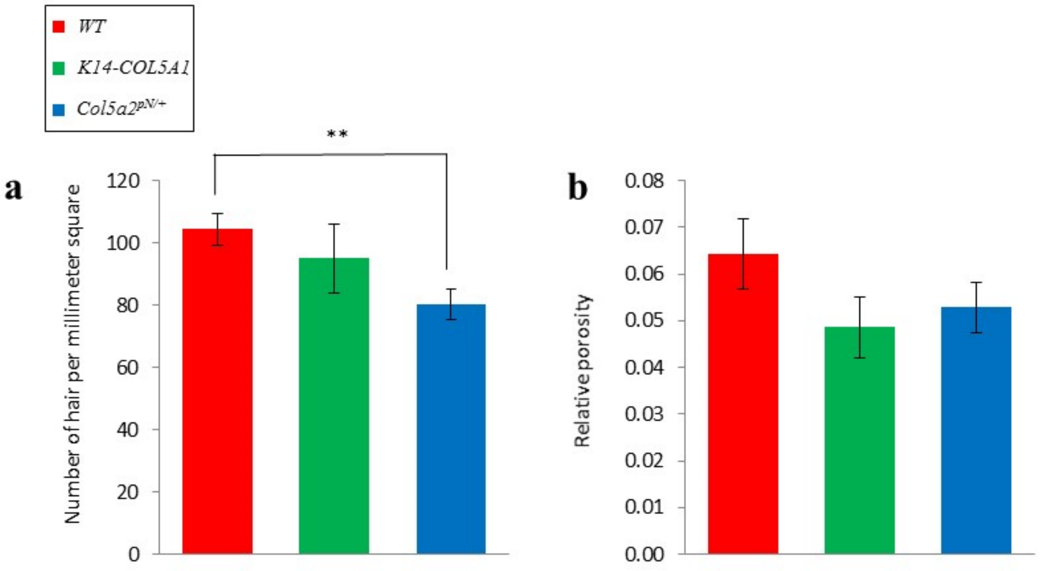

Supplementary Figure S6: Number of hair and average porosity per surface in *WT*, *K14-COL5A1*, and *Col5a2*$^{pN/+}$ murine skin.

(**a**) Number of hair per millimiter square; (**b**) Porosity per millimiter square. Error bars corresponds to SEM. Averaged values only over samples with SHG analysis. * (resp. **) indicates $p<0.05$ (resp $p<0.01$).

Supplementary Tables

Supplementary Table S1: Relationship between local and global stretch ratio for *WT* skin samples.

Linear fitting of the variation of the local deformation tensor as a function of the global stretch ratio (see Figures 3.a and 3.b) provides 2 parameters: the intercept $\lambda_{ii,1}$ and the slope A_{ii}, with squared correlation coefficient R^2. These parameters are averaged over the control murine populations (± SEM). Wild-type mice samples show consistent results, with a slope in the traction direction equal to 1, indicating that the global stretch is fully transmitted to the local scale. * stands for: no complete mechanical analysis, # for: no complete SHG analysis. Wild-type specimens are obtained either from 129sv strains (*WT*), or by crossing and genotyping transgenic mice *K14-COL5A1 (K14-COL5A1^{WT})* or *Col5a2$^{pN/+}$* heterozygous mice (*Col5a2$^{+/+}$*).

Type	No	λ_{xx}			λ_{yy}		
		$\lambda_{xx,1}$	A_{xx}	R^2	$\lambda_{yy,1}$	A_{yy}	R^2
WT	*WT 4*	0.992 ± 0.004	1.13 ± 0.03	0.99	1.018 ± 0.002	-0.55 ± 0.02	0.99
	WT 5	0.975 ± 0.008	1.04 ± 0.03	0.99	1.42 ± 0.03	-0.97 ± 0.08	0.97
	WT6	0.91 ± 0.01	0.89 ± 0.04	0.98	1.39 ± 0.09	-0.9 ± 0.3	0.75
	K14-COL5A1^{WT} 92	1.00 ± 0.01	1.0 ± 0.1	0.89	1.46 ± 0.03	-1.46 ± 0.07	0.99
	K14-COL5A1^{WT} 93	0.89 ± 0.02	0.78 ± 0.06	0.94	1.06 ± 0.02	-0.90 ± 0.06	0.96
	K14-COL5A1^{WT} 21	0.97 ± 0.01	1.17 ± 0.04	0.99	1.54 ± 0.04	-1.8 ± 0.1	0.98
	K14-COL5A1^{WT} 22	0.985 ± 0.004	1.11 ± 0.02	0.99	1.32 ± 0.01	-1.33 ± 0.05	0.99
	K14-COL5A1^{WT} 25	1.023 ± 0.008	0.90 ± 0.02	0.99	1.48 ± 0.03	-0.92 ± 0.07	0.96
	K14-COL5A1^{WT} 26	1.00 ± 0.02	0.7 ± 0.1	0.88	1.22 ± 0.02	-1.6 ± 0.1	0.98
	K14-COL5A1^{WT} 35 #	/	/	/	/	/	/
	Col5a2$^{+/+}$ 70	1.01 ± 0.01	0.99 ± 0.03	0.99	1.80 ± 0.08	-1.1 ± 0.1	0.93
	Col5a2$^{+/+}$ 72	0.96 ± 0.02	1.26 ± 0.08	0.97	1.26 ± 0.01	-1.61 ± 0.04	0.99
	Col5a2$^{+/+}$ 74	1.02 ± 0.02	1.06 ± 0.07	0.97	1.55 ± 0.06	-1.9 ± 0.2	0.95
	Col5a2$^{+/+}$ 76	0.97 ± 0.01	1.17 ± 0.06	0.98	1.35 ± 0.01	-1.77 ± 0.05	0.99
	Col5a2$^{+/+}$ 77	0.97 ± 0.01	1.33 ± 0.07	0.98	1.50 ± 0.02	-1.9 ± 0.1	0.99
	Col5a2$^{+/+}$ 78	1.05 ± 0.02	1.37 ± 0.08	0.97	1.31 ± 0.03	-2.0 ± 0.1	0.98
	Col5a2$^{+/+}$ 79	0.80 ± 0.03	2.1 ± 0.1	0.98	1.26 ± 0.03	-2.2 ± 0.1	0.99
	Col5a2$^{+/+}$ 80	0.96 ± 0.07	1.8 ± 0.4	0.79	1.15 ± 0.02	-1.74 ± 0.09	0.99
	Col5a2$^{+/+}$ 82-12	1.09 ± 0.01	1.97 ± 0.05	0.99	1.51 ± 0.05	-1.65 ± 0.01	0.95
	Col5a2$^{+/+}$ 84-13	0.94 ± 0.03	0.7 ± 0.1	0.86	1.38 ± 0.04	-1.6 ± 0.1	0.97
	Col5a2$^{+/+}$ 85	1.01 ± 0.01	0.86 ± 0.06	0.97	1.08 ± 0.01	-0.86 ± 0.08	0.96
	Col5a2$^{+/+}$ 86	0.97 ± 0.02	0.76 ± 0.07	0.94	1.53 ± 0.04	-2.0 ± 0.1	0.98
	Col5a2$^{+/+}$ 87	0.97 ± 0.02	0.9 ± 0.1	0.91	1.43 ± 0.02	-2.37 ± 0.09	0.99
	Col5a2$^{+/+}$ 88	1.03 ± 0.02	0.51 ± 0.07	0.84	1.28 ± 0.03	-1.77 ± 0.08	0.99
	Col5a2$^{+/+}$ 90	1.01 ± 0.05	1.00 ± 0.04	0.99	-0.4 ± 0.1	-1.7 ± 0.1	0.97
**Average**		0.98 ± 0.01	1.10 ± 0.08		1.29 ± 0.08	-1.5 ± 0.1	

Supplementary Table S2: Relationship between local and global stretch ratio for *K14-COL5A1* and *Col5a2$^{pN/+}$* skin samples.

Linear fitting of the variation of the local deformation tensor as a function of the global stretch ratio (see Figures 3.a and 3.b) provides 2 parameters: the intercept $\lambda_{ii,1}$ and the slope A_{ii}, with squared correlation coefficient R^2. These parameters are averaged over the transgenic *K14-COL5A1* and heterozygous *Col5a2$^{pN/+}$* murine populations (± SEM). Both mice models show consistent results, with a slope in the traction direction equal to 1, indicating that the global stretch is fully transmitted to the local scale. * stands for: no complete mechanical analysis, # for: no complete SHG analysis.

Type	No	λ_{xx}			λ_{yy}		
		$\lambda_{xx,1}$	A_{xx}	R^2	$\lambda_{yy,1}$	A_{yy}	R^2
K14-COL5A1	*91*	1.00 ± 0.02	1.06 ± 0.04	0.99	1.270 ± 0.004	-0.90 ± 0.01	0.99
	94	1.00 ± 0.01	1.1 ± 0.2	0.85	1.32 ± 0.03	-1.2 ± 0.1	0.97
	24	0.982 ± 0.006	0.85 ± 0.02	0.99	1.28 ± 0.03	-0.71 ± 0.09	0.94
	29	1.02 ± 0.02	0.98 ± 0.06	0.97	1.47 ± 0.06	-1.5 ± 0.2	0.94
	30	1.00 ± 0.01	1.11 ± 0.04	0.99	1.40 ± 0.04	-1.2 ± 0.1	0.96
	32	0.98 ± 0.01	1.06 ± 0.03	0.99	1.36 ± 0.02	-0.89 ± 0.05	0.99
	37	1.00 ± 0.01	1.29 ± 0.06	0.99	1.30 ± 0.01	-0.88 ± 0.05	0.99
	38	1.059 ± 0.006	1.14 ± 0.02	0.99	1.42 ± 0.04	-1.1 ± 0.1	0.95
	39	0.95 ± 0.01	1.53 ± 0.07	0.99	1.36 ± 0.06	-0.9 ± 0.2	0.87
**Average**		0.999 ± 0.010	1.12 ± 0.06		1.35 ± 0.02	-1.03 ± 0.08	
Col5a2$^{pN/+}$	*81-12*	1.039 ± 0.007	1.05 ± 0.02	0.99	1.25 ± 0.02	-1.00 ± 0.05	0.99
	*84-12 **	1.0 ± 0.1	1.34 ± 0.04	0.99	1.11 ± 0.02	-0.46 ± 0.04	0.93
	73	0.95 ± 0.02	1.37 ± 0.09	0.97	1.31 ± 0.03	-2.0 ± 0.1	0.98
	71	0.96 ± 0.03	1.1 ± 0.1	0.90	1.28 ± 0.01	-2.12 ± 0.06	0.99
	81-13	1.04 ± 0.05	0.6 ± 0.2	0.54	1.88 ± 0.07	-2.0 ± 0.1	0.97
	75	0.99 ± 0.01	0.71 ± 0.04	0.98	1.26 ± 0.01	-1.58 ± 0.04	0.99
	82-13	0.96 ± 0.02	1.0 ± 0.1	0.91	1.24 ± 0.06	-1.3 ± 0.3	0.88
	83 #	/	/	/	1.58 ± 0.03	-1.57 ± 0.09	0.98
	33 #	/	/	/	/	/	/
	38	0.9 ± 0.1	1.2 ± 0.1	0.98	1.06 ± 0.02	-1.85 ± 0.02	0.99
	39	1.0 ± 0.1	0.9 ± 0.1	0.95	1.08 ± 0.07	-1.71 ± 0.06	0.96
	40	1.0 ± 0.2	0.8 ± 0.2	0.99	1.06 ± 0.03	-1.55 ± 0.03	0.99
	42 #	0.9 ± 0.3	1.2 ± 0.2	0.97	1.14 ± 0.06	-1.76 ± 0.05	0.93
	45	0.9 ± 0.2	1.4 ± 0.2	0.89	1.15 ± 0.05	-2.00 ± 0.04	0.97
	48	1.0 ± 0.1	1.4 ± 0.1	0.99	1.06 ± 0.09	-1.90 ± 0.08	1.00
	*49 **	1.0 ± 0.1	1.13 ± 0.08	0.99	1.18 ± 0.10	-1.69 ± 0.08	0.93
	51	1.00 ± 0.03	0.84 ± 0.03	1.00	1.02 ± 0.02	-0.97 ± 0.02	1.00
**Average**		0.97 ± 0.01	1.07 ± 0.06		1.23 ± 0.06	-1.6 ± 0.1	

Supplementary Table S3: Collagen reorganization upon stretching for *WT* skin samples.

Linear fitting of the OI variation (resp. the entropy variation) as a function of the global stretch (see Figures 3.e and 3.f) provides 2 parameters: the intercept OI(1) (resp. S(1)) and the slope A_{OI} (resp. A_S), with squared correlation coefficient R^2 (fitting quality). These parameters are averaged over the WT murine populations (± SEM), which exhibit different behavior. * stands for: no complete mechanical analysis, # for: no complete SHG analysis. Wild-type specimens are obtained either from 129sv strains (WT), or by crossing and genotyping transgenic mice *K14-COL5A1 (K14-COL5A1^{WT})* or *Col5a2$^{pN/+}$* heterozygous mice (*Col5a2$^{+/+}$*).

	No	OI			S		
		OI(1)	A_{OI}	R^2	S(1)	A_S	R^2
**WT**	*WT 4*	5.9 ± 0.7	88 ± 6	0.98	2.992 ± 0.002	-0.26 ± 0.02	0.98
	WT 5	-5 ± 2	62 ± 5	0.96	3.04 ± 0.01	-0.25 ± 0.03	0.93
	WT 6	15.7 ± 0.9	47 ± 2	0.99	3.008 ± 0.008	-0.36 ± 0.02	0.99
	K14-COL5A1^{WT} 92	-3 ± 2	85 ± 4	0.98	3.471 ± 0.009	-0.45 ± 0.03	0.98
	K14-COL5A1^{WT} 93	8.2 ± 0.8	88 ± 3	0.94	3.424 ± 0.005	-0.51 ± 0.02	0.99
	K14-COL5A1^{WT} 21	-4.9 ± 0.9	90 ± 3	0.99	3.066 ± 0.008	-0.43 ± 0.02	0.98
	K14-COL5A1^{WT} 22	4.8 ± 0.7	82 ± 3	0.99	3.015 ± 0.004	-0.37 ± 0.02	0.99
	K14-COL5A1^{WT} 25	-3.1 ± 0.5	60 ± 1	0.99	3.051 ± 0.008	-0.28 ± 0.02	0.97
	K14-COL5A1^{WT} 26	8.8 ± 0.8	75 ± 3	0.99	3.004 ± 0.005	-0.40 ± 0.02	0.98
	K14-COL5A1^{WT} 35 #	1 ± 2	72 ± 6	0.96	3.034 ± 0.007	-0.35 ± 0.02	0.98
	Col5a2$^{+/+}$ 70	-11.0 ± 0.9	65 ± 2	0.99	2.981 ± 0.006	-0.29 ± 0.01	0.99
	Col5a2$^{+/+}$ 72	14.9 ± 0.4	76 ± 2	0.99	2.896 ± 0.009	-0.54 ± 0.04	0.98
	Col5a2$^{+/+}$ 74	8.4 ± 0.9	70 ± 3	0.99	2.93 ± 0.01	-0.44 ± 0.04	0.96
	Col5a2$^{+/+}$ 76	7.5 ± 0.7	87 ± 3	0.99	2.916 ± 0.006	-0.49 ± 0.02	0.99
	Col5a2$^{+/+}$ 77	12 ± 1	75 ± 4	0.99	2.922 ± 0.006	-0.56 ± 0.02	0.99
	Col5a2$^{+/+}$ 78	5 ± 2	108 ± 6	0.99	2.93 ± 0.01	-0.58 ± 0.05	0.97
	Col5a2$^{+/+}$ 79	3 ± 2	101 ± 7	0.97	2.96 ± 0.01	-0.65 ± 0.04	0.98
	Col5a2$^{+/+}$ 80	18.1 ± 0.4	75 ± 2	0.99	2.869 ± 0.005	-0.50 ± 0.02	0.99
	Col5a2$^{+/+}$ 82-12	16.40 ± 0.07	61 ± 4	0.97	3.377 ± 0.002	-0.35 ± 0.01	0.99
	Col5a2$^{+/+}$ 84-13	-2 ± 1	94 ± 4	0.99	2.953 ± 0.006	-0.48 ± 0.02	0.99
	Col5a2$^{+/+}$ 85	15.4 ± 0.3	70 ± 1	1.00	2.903 ± 0.005	-0.53 ± 0.02	1.00
	Col5a2$^{+/+}$ 86	-1.40 ± 0.05	113 ± 2	1.00	2.965 ± 0.009	-0.64 ± 0.03	0.99
	Col5a2$^{+/+}$ 87	14.30 ± 0.08	75 ± 3	1.00	2.907 ± 0.003	-0.55 ± 0.01	1.00
	Col5a2$^{+/+}$ 88	13 ± 1	77 ± 4	0.98	2.91 ± 0.01	-0.56 ± 0.04	0.97
	Col5a2$^{+/+}$ 90	20 ± 4	83 ± 3	0.99	2.86 ± 0.05	-0.56 ± 0.04	0.98
**Average**		6 ± 2	79 ± 3		3.02 ± 0.03	-0.46 ± 0.02	

Supplementary Table S4: Collagen reorganization upon stretching for *K14-COL5A1* and *Col5a2$^{pN/+}$* skin samples.

Linear fitting of the OI variation (resp. the entropy variation) as a function of the global stretch (see Figures 3.e and 3.f) provides 2 parameters: the intercept OI(1) (resp. S(1)) and the slope A_{OI} (resp. A_S), with squared correlation coefficient R^2 (fitting quality). These parameters are averaged over the transgenic *K14-COL5A1* and heterozygous *Col5a2$^{pN/+}$* murine populations (± SEM), which exhibit different behavior. * stands for: no complete mechanical analysis, # for: no complete SHG analysis.

Type	No	OI			S		
		OI(1)	A_{OI}	R^2	S(1)	A_S	R^2
K14-COL5A1	*91*	13.5 ± 0.5	52 ± 1	0.99	3.384 ± 0.007	-0.277 ± 0.008	0.99
	94	4 ± 1	80 ± 3	0.99	3.452 ± 0.007	-0.49 ± 0.02	0.97
	24	7.2 ± 0.8	58 ± 2	0.98	3.04 ± 0.01	-0.38 ± 0.03	0.96
	29	0 ± 3	64 ± 9	0.89	3.03 ± 0.01	-0.26 ± 0.05	0.84
	30	-1.1 ± 0.8	57 ± 2	0.99	3.038 ± 0.004	-0.247 ± 0.009	0.99
	32	-4.40 ± 0.03	64 ± 1	0.99	3.033 ± 0.004	-0.22 ± 0.01	0.99
	37	0.8 ± 0.5	63 ± 2	0.99	3.025 ± 0.007	-0.26 ± 0.02	0.96
	38	-7 ± 1	67 ± 1	0.98	3.073 ± 0.006	-0.31 ± 0.01	0.99
	39	7.9 ± 0.9	30 ± 4	0.86	2.986 ± 0.003	-0.08 ± 0.02	0.77
**Average**		2 ± 2	59 ± 5		3.12 ± 0.06	-0.28 ± 0.04	
Col5a2$^{pN/+}$	*81-12*	8.1 ± 0.5	52 ± 2	0.99	3.574 ± 0.002	-0.20 ± 0.01	0.96
	*84-12 **	16.3 ± 0.7	47 ± 1	0.99	3.409 ± 0.008	-0.36 ± 0.02	0.98
	73	5 ± 2	81 ± 5	0.98	2.96 ± 0.02	-0.55 ± 0.07	0.93
	71	17.4 ± 0.9	79 ± 4	0.98	2.875 ± 6E-04	-0.55 ± 0.03	0.98
	81-13	9 ± 2	44 ± 4	0.90	2.963 ± 0.008	-0.37 ± 0.02	0.98
	75	14.2 ± 0.6	67 ± 2	0.99	2.901 ± 0.005	-0.48 ± 0.01	0.99
	82-13	10.8 ± 0.9	71 ± 5	0.98	2.901 ± 0.008	-0.40 ± 0.04	0.96
	83 #	3.6 ± 0.5	29 ± 2	0.98	2.891 ± 0.002	-0.066 ± 0.006	0.96
	33 #	16 ± 5	52 ± 4	0.96	2.86 ± 0.02	-0.25 ± 0.02	0.96
	38	26 ± 2	45 ± 2	0.99	2.83 ± 0.03	-0.31 ± 0.03	0.96
	39	22 ± 5	84 ± 5	0.98	2.84 ± 0.03	-0.59 ± 0.03	0.99
	40	10 ± 5	76 ± 4	0.99	2.88 ± 0.04	-0.29 ± 0.03	0.95
	42 #	/	/	/	/	/	/
	48	23 ± 8	54 ± 7	0.91	2.85 ± 0.04	-0.42 ± 0.03	0.96
	*49 **	18 ± 8	89 ± 7	0.97	2.87 ± 0.03	-0.57 ± 0.03	0.99
	51	19 ± 3	54 ± 3	0.98	2.85 ± 0.04	-0.33 ± 0.03	0.93
	92	39 ± 4	27 ± 3	0.91	2.73 ± 0.04	-0.26 ± 0.03	0.92
**Average**		16 ± 2	60 ± 5		2.95 ± 0.06	-0.38 ± 0.04	

Supplementary Table S5: Mechanical data for *WT* skin samples.

The stress/stretch response is characterized by 4 parameters: the length of the heel region, the ultimate stretch ratio, the ultimate tensile strength and the tangent modulus that corresponds to the slope of the linear part obtained by linear fitting with squared correlation coefficient R^2. These parameters are averaged over the WT murine populations (± SEM). * stands for: no complete mechanical analysis, # for: no complete SHG analysis. Wild-type specimens are obtained either from 129sv strains (WT), or by crossing and genotyping transgenic mice *K14-COL5A1 (K14-COL5A1^{WT})* or *Col5a2$^{pN/+}$* heterozygous mice (*Col5a2$^{+/+}$*).

	No	Sample size (mm)			Stress/stretch				
		Length	Width	Thickness	Heel region length	Ultimate stretch ratio	Ultimate tensile strength (MPa)	Tangent modulus (MPa)	R^2
WT	*WT 4*	15.0	10.0	1.0	0.13	NA	NA	2.27	0.98
	WT 5	16.3	8.5	1.0	0.16	1.39	0.37	1.39	0.99
	WT6	18.7	8.1	0.8	0.22	1.47	0.66	2.09	0.99
	K14-COL5A1^{WT} 92	22.1	10.7	0.9	0.28	1.49	0.26	0.90	0.99
	K14-COL5A1^{WT} 93	19.9	11.1	1.1	0.24	1.48	0.36	1.00	0.99
	K14-COL5A1^{WT} 21	19.1	7.5	1.0	NA	1.46	0.58	1.60	0.98
	K14-COL5A1^{WT} 22	17.9	7.9	0.8	0.14	1.41	0.57	2.28	0.99
	K14-COL5A1^{WT} 25	16.7	8.2	1.0	0.18	1.54	0.56	1.71	0.99
	K14-COL5A1^{WT} 26	25.0	7.5	1.2	0.11	1.39	0.55	1.70	0.94
	K14-COL5A1^{WT} 35 #	18.0	9.9	1.2	NA	1.47	0.30	0.86	0.97
	Col5a2$^{+/+}$ 70	18.9	5.8	1.1	0.20	1.28	0.47	3.29	0.99
	Col5a2$^{+/+}$ 72	25.2	8.7	1.3	0.16	1.41	0.46	1.59	0.99
	Col5a2$^{+/+}$ 74	25.5	7.0	1.4	0.12	1.45	0.56	1.71	0.99
	Col5a2$^{+/+}$ 76	28.8	9.1	1.5	0.14	1.42	0.30	0.98	0.98
	Col5a2$^{+/+}$ 77	25.8	8.3	1.4	0.16	1.41	0.31	1.26	0.99
	Col5a2$^{+/+}$ 78	25.1	6.8	1.0	0.08	1.40	0.68	2.38	0.99
	Col5a2$^{+/+}$ 79	26.5	7.0	1.3	0.08	1.38	0.45	1.94	0.99
	Col5a2$^{+/+}$ 80	21.9	8.1	1.1	0.09	1.33	0.18	0.88	0.99
	Col5a2$^{+/+}$ 82-12	17.2	10.1	1.1	NA	1.44	0.31	0.75	0.98
	Col5a2$^{+/+}$ 84-13	23.6	10.1	1.0	0.09	1.47	0.32	1.27	0.99
	Col5a2$^{+/+}$ 85	17.4	7.6	1.2	0.15	1.49	0.40	1.22	0.99
	Col5a2$^{+/+}$ 86	23.4	7.3	0.9	0.14	1.44	0.49	1.67	0.99
	Col5a2$^{+/+}$ 87	26.1	4.9	0.8	0.10	1.42	0.90	2.34	0.99
	Col5a2$^{+/+}$ 88	21.6	6.5	1.1	0.13	1.46	0.42	1.55	0.99
	Col5a2$^{+/+}$ 90	16.2	4.8	0.9	NA	1.34	0.76	3.11	0.99
	K14-COL5A1^{WT} 36 #	16.6	8.3	0.8	0.18	1.61	0.55	0.92	0.99
	K14-COL5A1^{WT} 51 #	13.7	7.5	0.5	NA	1.43	0.49	1.48	0.99
	K14-COL5A1^{WT} 52 #	17.1	6.1	0.9	NA	1.41	0.45	2.72	0.99
	K14-COL5A1^{WT} 53 #	17.5	7.2	0.7	NA	1.38	0.67	2.20	0.99
	K14-COL5A1^{WT} 54 #	14.1	9.1	0.5	0.13	1.52	0.66	1.00	0.99
	K14-COL5A1^{WT} 63 #	22.7	6.7	0.7	0.16	1.22	0.16	1.25	0.99
	K14-COL5A1^{WT} 95 #	25.2	10.9	1.2	0.10	1.45	0.32	0.76	0.99
**Average**		20.6 ± 0.7	8.0 ± 0.3	1.0 ± 0.04	0.15 ± 0.01	1.43 ± 0.01	0.47 ± 0.03	1.63 ± 0.12	

Supplementary Table S6: Mechanical data for *K14-COL5A1* and *Col5a2*$^{pN/+}$ skin samples.

The stress/stretch response is characterized by 4 parameters: the length of the heel region, the ultimate stretch ratio, the ultimate tensile strength and the tangent modulus that corresponds to the slope of the linear part obtained by linear fitting with squared correlation coefficient R^2. These parameters are averaged over the transgenic *K14-COL5A1* and heterouzygous *Col5a2*$^{pN/+}$ murine populations (± SEM). * stands for: no complete mechanical analysis, # for: no complete SHG analysis.

Type	No	Sample size (mm)			Stress/stretch				
		Length	Width	Thickness	Heel region length	Ultimate stretch ratio	Ultimate tensile strength (MPa)	Tangent modulus (MPa)	R^2
K14-COL5A1	*91*	17.2	9.1	1.1	0.24	1.46	0.38	1.22	0.99
	94	19.4	9.0	1.1	0.24	1.41	0.24	1.14	0.99
	24	16.2	4.8	1.1	0.30	1.45	0.43	2.31	0.99
	29	17.0	9.6	1.0	0.14	1.41	0.47	2.19	0.99
	30	15.7	9.1	0.7	0.20	1.50	0.75	2.84	0.99
	32	15.4	8.1	0.9	0.21	1.48	0.88	2.91	0.99
	37	15.1	9.7	1.0	0.21	1.42	0.62	2.33	0.99
	38	16.2	9.3	0.9	0.12	1.41	0.60	2.31	0.99
	39	15.3	9.4	0.8	0.18	1.32	0.73	3.36	0.99
	44 #	12.3	6.1	0.9	0.27	1.52	0.75	2.40	0.99
	45 #	14.7	5.7	1.0	0.17	1.46	0.91	2.67	0.99
	46 #	12.4	6.1	0.7	0.26	1.43	0.68	3.32	0.99
	48 #	15.9	5.2	1.0	0.25	1.67	0.44	1.85	0.99
	49 #	12.6	10.5	0.8	0.32	1.54	0.55	2.36	0.99
	50 #	13.5	5.5	0.6	0.23	1.43	1.12	4.30	0.99
	55 #	18.0	9.2	0.8	0.23	1.30	0.36	2.67	0.99
**Average**		15.4 ± 0.5	7.9 ± 0.5	0.9 ± 0.04	0.22 ± 0.01	1.45 ± 0.02	0.62 ± 0.06	2.51 ± 0.195	
Col5a2$^{pN/+}$	*81-12*	17.8	8.9	1.1	0.13	1.58	0.60	1.56	0.99
	*84-12 **	19.9	10.7	1.2	/	/	/	/	/
	73	19.8	7.2	1.1	0.12	1.47	1.40	2.75	0.97
	71	24.5	8.1	1.4	0.10	1.40	0.31	0.98	0.97
	81-13	16.1	6.8	1.2	0.17	1.51	0.41	1.10	0.98
	75	19.7	8.8	1.2	0.14	1.41	0.32	1.15	0.99
	82-13	23.6	8.8	1.1	0.13	1.41	0.32	1.02	0.99
	83	26.1	7.5	1.2	0.26	1.41	0.25	1.14	0.99
	33 #	29.6	6.2	1.0	0.10	1.37	0.66	1.15	0.99
	38 #	31.2	5.1	0.6	0.15	1.37	0.39	3.12	0.99
	39	23.3	4.6	0.7	0.14	1.31	0.79	2.05	0.99
	40	27.8	4.6	0.9	0.15	1.34	0.54	1.97	0.99
	42 #	28.2	4.1	0.6	0.09	1.32	0.53	3.20	0.98
	45	32.6	7.9	0.9	0.05	1.16	0.80	1.57	0.99
	48	30.0	3.3	0.6	0.06	1.36	0.19	3.90	0.99
	*49 **	28.0	7.4	0.8	/	/	/	/	/
	51	21.2	4.4	0.8	0.21	1.36	1.11	1.66	0.99
	92	17.0	7.6	0.7	0.18	1.30	0.39	3.51	0.99
**Average**		24.2 ± 1.2	6.8 ± 0.5	1.0 ± 0.1	0.14 ± 0.01	1.38 ± 0.02	0.58 ± 0.08	2.0 ± 0.25	
